# Extracellular Vesicles in Cancer: Mechanistic Insights and Clinical Applications

**DOI:** 10.3390/cancers18030537

**Published:** 2026-02-06

**Authors:** Fahad A. Alhumaydhi, Shehwaz Anwar

**Affiliations:** 1 Department of Medical Laboratories, College of Applied Medical Sciences, Qassim University, Buraydah 51452, Saudi Arabia; f.alhumaydhi@qu.edu.sa; 2 Department of Medical Laboratory Technology, Mohan Institute of Nursing and Paramedical Sciences, Bareilly 243302, India

**Keywords:** extracellular vesicles, cancer, intercellular communication, liquid biopsy, biomarkers, drug delivery

## Abstract

Extracellular vesicles (EVs) have emerged as important messengers in cell-to-cell communication, carrying biologically active molecules such as lipids, nucleic acids, and proteins that influence both normal physiology and disease. In cancer, EVs play complex and context-dependent roles, contributing to tumor growth, angiogenesis, immune evasion, metastasis, and resistance to therapy, while in certain settings, they may also support antitumor immune responses. Increasing evidence shows that EVs released from tumor and stromal cells actively reshape the tumor microenvironment (TME) and participate in the formation of pre-metastatic niches, thereby facilitating cancer dissemination. Because EVs are stable, readily detectable in body fluids, and reflect the molecular characteristics of their cells of origin, they have attracted considerable interest as minimally invasive biomarkers for cancer diagnosis, prognosis, and treatment monitoring. In addition, their natural biocompatibility makes them attractive candidates for targeted drug delivery. This review summarizes current knowledge on EV biogenesis, cargo composition, and functional roles in cancer progression, with a particular focus on recent advances in their clinical applications. Key challenges related to EV isolation, characterization, and clinical translations are also discussed, highlighting future opportunities for integrating EV-based strategies into precision oncology.

## Simple Summary

Extracellular vesicles (EVs) are increasingly recognized as critical regulators of cancer biology, facilitating intercellular communication through the transfer of bioactive cargo, such as nucleic acids, proteins, and lipids. EVs derived from tumor cells and components of the tumor microenvironment (TME) influence multiple hallmarks of cancer, including angiogenesis, immune modulation, extracellular matrix remodeling, metastatic niche formation, and therapeutic resistance. Their capacity to reflect tumor-specific molecular alterations and remain stable in body fluids highlights their potential as minimally invasive biomarkers for cancer detection and disease monitoring. Moreover, advances in EV engineering have opened new opportunities for their use as targeted drug delivery systems and immunotherapeutic platforms. Despite these advances, challenges related to EV heterogeneity, isolation methods, scalability, and clinical standardization continue to limit translation into routine oncology practices.

## 1. Introduction

Due to its increasing incidence and mortality in recent years, cancer continues to rank among the world’s leading causes of death despite advances in therapeutic approaches, positioning it as a major global health burden [1]. Cancer is associated with high rates of mortality and morbidity [2,3]. Cancer-related mortality currently affects about 10 million individuals per year and is projected to increase markedly by 2030 [4]. Cancer progression and therapeutic response are strongly influenced by multifaceted relationships across malignant cells and the tumor microenvironment (TME), comprising stromal and immune components, while genetic, epigenetic, and environmental factors collectively drive tumor heterogeneity and treatment resistance [5].

Surgery, chemotherapy, and radiotherapy are still the principal primary therapeutic choices for individuals confirmed at various stages of cancer [6]. However, the adverse effects associated with chemotherapy vary depending on the organs and systems involved, often limiting the dose that can be safely administered and thereby reducing therapeutic efficacy [7]. Moreover, existing strategies aimed at mitigating chemotherapy-induced toxicity frequently cause additional adverse effects, rendering them insufficient. Consequently, there is a critical need to reduce treatment-related toxicity in order to maintain optimal dosing. The development of safer, better targeted, and more efficacious treatment approaches is therefore essential for improving cancer patient outcomes [8,9]. Recent advances have led to the development of novel therapeutic strategies, facilitating the improved identification of molecular markers and new treatment targets [9].

Timely identification of cancer through screening of both high-risk and apparently healthy populations significantly improves the likelihood of successful intervention and reduces mortality. At present, imaging modalities are widely used for the diagnosis of most solid tumors, with confirmation typically achieved through tissue biopsy. However, biopsy-derived information may not accurately represent tumor heterogeneity due to sampling limitations related to the site of tissue collection, thereby restricting its ability to reliably assess treatment efficacy or detect disease progression [10,11]. In contrast, liquid biopsy and blood-based biomarkers may point to genetic alterations associated with tumors and identify acquired therapeutic resistance or disease recurrence before symptoms develop [10,11]. Nevertheless, conventional tumor markers exhibit significant limitations and are often inadequate for routine clinical application [10,12]. Innovative, non-invasive diagnostic techniques that can accurately monitor treatment response, enable early cancer identification, and fully characterize tumor features are therefore desperately needed [10,11]. Liquid biopsy approaches, compared with traditional tissue biopsy, offer distinct advantages in detecting disease recurrence and uncovering tumor-associated genetic changes, thereby improving clinical decision-making [12,13].

Liquid biopsy is a popular noninvasive diagnostic approach that analyzes circulating tumor-derived components found in bodily fluids. Among these components, extracellular vesicles (EVs) have emerged as a major focus of research over the past few years. EVs carry tumor-associated DNA, coding and non-coding RNAs, and proteins, underscoring their promise for early cancer diagnosis, prognosis, and therapeutic monitoring [12,14]. This review outlines the evolving significance of EVs in cancer diagnosis and therapy, emphasizing their biological characteristics, roles in tumor progression, and clinical relevance as diagnostic biomarkers and therapeutic tools. In addition, recent technological advances, ongoing clinical applications, and major challenges limiting the translational use of EVs-based strategies are addressed.

## 2. Biology of Extracellular Vesicles (EVs)

EVs represent lipid bilayer-encapsulated particles actively released by a wide range of cells into the extracellular space. EVs transport diverse cargo, including extracellular matrix components, transcription factors, signaling proteins, receptors, and enzymes. In addition, EVs transport nucleic acids and lipids from originating cells to target cells, facilitating molecular signaling and intercellular communication. Accumulating evidence links EVs to numerous pathological conditions, such as cancer, cardiovascular disease, and neurodegenerative disorders. EVs function as key mediators of various intercellular communications, affecting numerous physiological and pathological processes [14].

All bodily fluids, such as plasma, urine, breast milk, saliva, and tears, contain EVs. Their widespread bio-distribution indicates both their capacity to influence diseased states and their function in normal physiological activity. The optimum methods for EV detection, purification, and analysis are still up for debate because the subject is still in its infancy. EVs are capable of traversing several physiological barriers, including the blood–brain barrier, thereby facilitating biomolecule transport and intercellular communication across distinct body compartments. Their effects are precise and targeted due to the cell-specific nature of their cargo, which makes it easier to fine-tune alterations in response to tissue state [15].

EVs are essential for many pathological processes, including tumorigenesis, growth, invasion, invasion, metastasis, aberrant neovascularization, epithelial–mesenchymal transition, immunological tolerance, immunological escape, and resistance to treatment [16]. Exosomes, often referred to as intraluminal vesicles or ILVs, are secreted by all cell types and protected by a single outer membrane [17].

Bioactive lipids, enzymes, and even chemical compounds called eicosanoids are carried by EVs and have chemotactic effects, which direct cell migration in a certain direction. Signaling chemicals called eicosanoids cause chemical gradients to form in bodily tissues. These gradients cause cells, especially immune cells, to move in the direction of the eicosanoid signal’s source. When immune cells are directed to areas of injury or infection by this chemotactic response, it plays a critical role in processes like inflammation. EVs provide vital interactions and reactions in the body’s immunological and healing processes by transferring these chemotactic chemicals [15].

Through several methods, cancer-derived EVs serve an important role in shaping the microenvironment that promotes tumor growth. EVs influence immune regulation by inducing apoptosis in dendritic cells and cytotoxic T cells, impairing NK cell activity and promoting the development of immunosuppressive cell populations such as myeloid-derived suppressor cells (MDSCs) and regulatory T cells (Treg cells). Moreover, they can alter the differentiation and polarization of various cells toward chemoresistant, immunosuppressive, and pro-tumorigenic states. Furthermore, EVs promote the polarization of fibroblasts into cancer-associated fibroblasts (CAFs), tumor-associated macrophages (TAMs) into M2 macrophages, and neutrophils into N2 phenotypes [18].

Tumor-derived EVs (T-EVs) mediate the transfer of bioactive cargo to tumor cells and tumor-associated cells including cancer stem cells (CSCs), fibroblasts, immune cells, and endothelial cells (EC). Conversely, EVs produced from non-tumor cells also affect the development of tumors in TME. Therefore, EV-mediated multidirectional signaling contributes to the complexity of TME [19].

EVs are secreted into the blood through cells of the peripheral nervous system and by neurons, microglia, astrocytes, and oligodendrocytes within the central nervous system (CNS). EVs are attractive and practical candidates for biomarker detection because they are easily accessible and can be readily obtained from the majority of bodily fluids. Additionally, EVs function as markers that reveal the pathophysiological state of the parent cells. Notably, the ability of EVs to traverse the blood–brain barrier highlights their promise as tools for diagnosing and treating neurological diseases [20].

EVs are grouped by size (large and small EVs), conceptual origin (oncosomes, migrasomes, stress EVs, and matrix vesicles), and production pathways (apoptosomes, exosomes, microvesicles, and autophagic EVs) [18]. EVs are operationally categorized into three major size-based populations including apoptotic bodies (1000–5000 nm), microvesicles (MVs) or ectosomes (100–1500 nm), and exosomes (30–100 nm) [15]. These EV populations exhibit distinct characteristics with respect to their biogenesis, secretion pathways, ranges of size, molecular cargo, and functions [16].

## 3. Sources of EVs

Blood is among the most intensively investigated sources of EVs in liquid biopsy research, as EVs circulate systemically and originate from different cells of multiple tissues. Their cellular origin reflects tissue heterogeneity and disease-associated alterations. The majority of EVs in the circulatory system come from platelets [21]. Platelets have a range of granules, and these granules are released in response to particular stimuli, resulting in the formation of MVs. Multiple cancer types, including gastric cancer (GC), lung cancer (LC), glioblastoma (GBM), and skin cancer (SC), have been reported to secrete EVs. The blood’s EV profile is also influenced by a wide range of immune cells, including monocytes, dendritic cells, macrophages, natural killer (NK) cells, megakaryocytes, B and T lymphocytes, and endothelial cells [22].

Adipocytes, muscular tissue, and cardiomyocytes, on the other hand, are comparatively conservative EV producers [23,24]. Interestingly, cancer cells are skilled at releasing EVs in large quantities into tissue fluids as well as the bloodstream. This feature increases EVs’ capacity for diagnosis and establishes them as useful indicators for the early identification and monitoring of a number of cancers and other medical disorders [22].

## 4. Biogenesis of EVs

**Figure 1:**
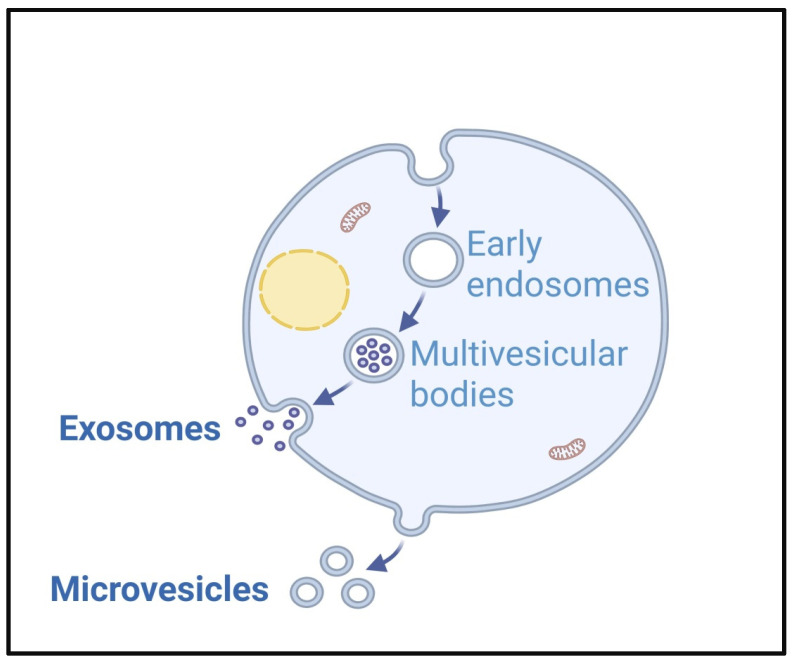
EV cargo, release, and biogenesis in cancer (created in https://BioRender.com). The process of exosome formation initiates with the invagination of the endosomal membrane, leading to early endosome formation. When the limiting membrane buds inward, intraluminal vesicles (ILVs) are produced and housed in a multivesicular body (MVB) or late endosome. Exosomes are then secreted into the extracellular environment following fusion of MVB bodies with the plasma membrane. The plasma membrane undergoes direct outward budding to produce microvesicles, commonly known as ectosomes (created in https://BioRender.com).

Although the precise mechanics are still mostly understood, there are a number of pathways for EV formation. Exosome formation involves sequential steps of endosomal membrane invagination, multivesicular body (MVB) transport, and subsequential release of exosomes. Each stage is controlled by distinct molecular regulators [25] (Figure 1).

Early and late phases of endosomes are membrane-bound vesicular compartments. Early endosomes are intracellular structures that are mostly found in the peripheral cytoplasm. After endocytosis, they receive and sift absorbed material. On the other hand, late endosomes are usually found closer to the nucleus and deeper into the cytoplasm. In contrast to lysosomes, endosomes are acidic vesicles devoid of lysosomal enzymes [26]. In the first step, the endosomal membrane pathway invades inward, forming the intraluminal vesicles inside MVBs [27]. After the formation, MVBs undergo directed intracellular trafficking to the plasma membrane, circumventing lysosomal degradation pathways. When MBVs fuse with the plasma membrane, intraluminal vesicles (ILVs) are released in the extracellular environment as exosomes [25].

Ectosomes, also known as MVs, are formed by outward protrusion and shedding of the plasma membrane. MV formation is also a multistep process involving membrane protrusion, budding, and scission. This process is driven by both lipid modeling and cytoskeletal dynamics. Membrane protrusion becomes initiated at actin enriched cellular structures like filopodia, lamellipodia, and membrane ruffles. This step is amplified in cancer by the aberrant activation of the Rho family GTPases (Rho, Rac1, Cdc42) through WASP/WAVE-mediated signaling, promoting enhanced membrane plasticity and directed motility. The redistribution of phosphatidylserine (PS) to the extracellular-facing layer of the plasma membrane generates local curvature, initiating vesicle budding. Final detachment of the membrane protrusions occurs through actin-myosin-driven contraction, releasing the ectosome into the extracellular space. In cancer, dysregulated actin dynamics alter the vesicle size, content, and function, enhancing their role in tumor invasion and metastasis [27]. While PS on EVs does signal macrophages to clear them, Flaskamp and colleagues showed that in vivo, many PS positive EVs remain on immune cells even if around half of them are cleared promptly. This implies that EVs may survive longer than expected, which is most likely due to tissue niches or protective surface proteins that delay their prompt phagocytosis [28].

In cancer, dysregulated actin dynamics can alter the vesicle size, cargo composition, and function, enhancing their role in tumor invasion and metastasis [25,26]. Although externalized phosphatidylserine is classically recognized as a phagocytic signal, its presence on extracellular vesicles does not necessarily lead to immediate clearance; vesicle fate is influenced by size, surface-associated proteins, and receptor-mediated uptake by specific target cells [15,16].

One important method of cell death for both healthy and malignant cells is apoptosis. The apoptotic process involves sequential morphological changes such as nuclear chromatin condensation, membrane blebbing, and the disassembly of the cell into membrane-enclosed apoptotic bodies. Apoptotic bodies arise solely during the execution of programmed cell death, in contrast to exosomes, microvesicles, and RLPs that are secreted under physiological cellular conditions. Although the presence of organelles within the vesicles characterizes apoptotic bodies, which are typically bigger in size, smaller vesicles are also released during this process. Whether membrane blebbing during apoptosis produced these tiny vesicles is still unknown [28].

Retrovirus-like particles, or RLPs, are EVs that mimic retroviral vesicles but lack the whole set of genes needed for viral multiplication or cellular entrance, making them non-infectious. Following a viral infection, cells release RLPs, which comprise a fraction of retroviral proteins. RLPs can be used by some viruses to help them spread and enter nearby cells [29]. RLPs emerge from the plasma membrane by budding straight. However, it is believed that the dynamics of plasma membranes associated with the generation of microvesicles or exosomes are different from the mechanism of biogenesis. Retroviral proteins, including Group-specific antigen (Gag), interact with cytoskeletal proteins and the plasma membrane component in the most commonly recognized mechanism for RLP production. Therefore, the Gag protein could be used as an RLP marker [28].

## 5. Methods of Isolation and Purification of EVs

The potential of extracellular vesicles for therapeutic application has not yet been matched by attempts to standardize the separation and purification processes [30]. Exosomal large-scale clinical practice use requires cheap cost, therapeutic efficacy, characterization, safety, high yield, high purity, ease of use, and speed [31]. Selecting an ideal, effective, and trustworthy isolation technique is one of the difficulties faced by broad application. The most popular isolation techniques are affinity separation, ultracentrifugation, size exclusion methods, precipitation, and microchips and nano-fluidics chips. Additionally, commercial kits are also used [32]. It takes a proper mix of isolation and purification techniques to separate healthy and well-purified extracellular vesicles [30].

Although basic differential ultracentrifugation is applied widely for concentrating EVs, advancements in the technique have been made to provide more pure formulations. Specifically, EVs are frequently separated from possible co-isolated pollutants using buoyant density centrifugation techniques (also referred to as isopycnic separation and zone centrifugation) that are tailored to their unique density (1.13–1.19 g/mL) [33]. Density gradient centrifugation has been shown to enhance high yield and greater purity compared to conventional ultracentrifugation [34,35]; however, this process is time-consuming, technically demanding, and limited to scalability. Additionally, processing clinical samples is not as well-suited for this procedure as processing huge volumes of samples. Crucially, EVs extracted by ultracentrifugation display compromised functional integrity [36] or a tendency to aggregate [37].

One of the most widely used techniques for isolating EVs based on size is ultrafiltration or microfiltration [32]. To rapidly and cost-effectively separate EVs from larger components, this approach employs membrane filtration using size-defined pore diameters (commonly 0.1, 0.22, or 0.45 μm) to filter EVs in suspension [38] or in conjunction with complementary EV isolation strategies [39].

Precipitation techniques are an additional option to ultracentrifugation. This method promotes vesicle aggregation through the addition of water-excluding polymers like polyethylene glycol (PEG) or lectins. These polymers reduce the solubility of vesicular components, thereby facilitating their precipitation by low-speed centrifugation [32].

As alternative approaches, the commercially available Total Exosome Isolation Reagent (ThermoFisher Scientific, Waltham, MA, USA) and the protein organic solvent precipitation (PROSPR) method [40] have been suggested as rapid and low-cost procedures for EV isolation [32]. Although biomarkers unique to EVs have not yet been established, several immunoaffinity-based methods have been introduced that exploit frequently expressed EV surface proteins and receptors [41,42,43,44]. These methods improve isolation performance by enhancing selectivity, yield, and vesicle integrity, particularly when processing complex or highly viscous biological samples, and they are readily compatible with established EV isolation workflows [45]. Immunoaffinity techniques are easy to implement and fast; however, their performance can be limited by antibody accessibility and heterogeneity in marker expression [46].

**Figure 2:**
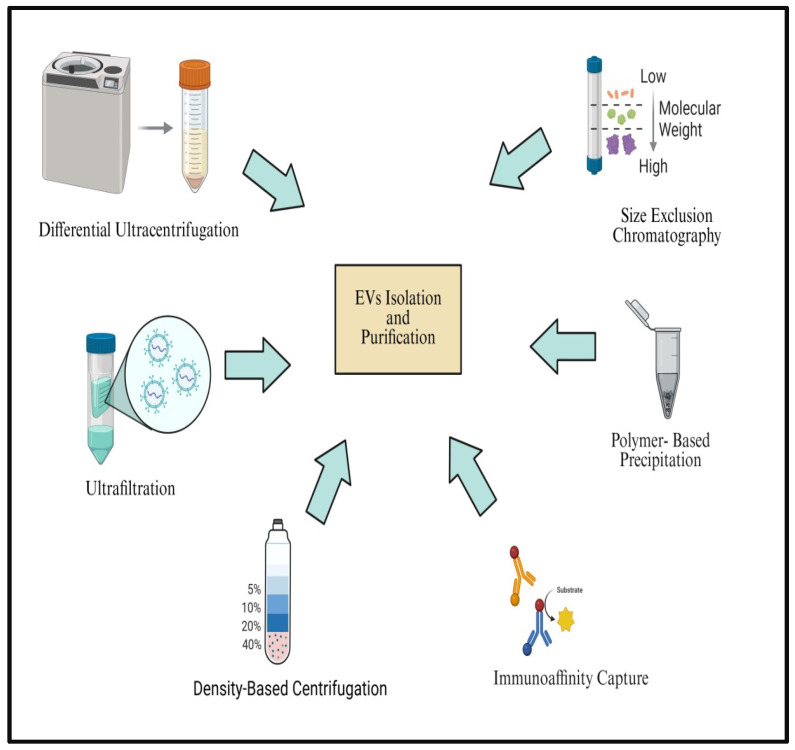
Major techniques used for the isolation and purification of EVs (created in https://BioRender.com). There are six major techniques for extracellular vesicle isolation and purification including differential ultracentrifugation, ultrafiltration, density-based centrifugation, polymer-based precipitation, size-exclusion chromatography, and immunoaffinity capture (created in https://BioRender.com).

Microfluidic-based technologies often combine high throughput with molecular detection capability and leverage the physical and biological characteristics of EVs at microscales [47,48]. Microfluidic EV separation strategies can be divided into size-based, immunoaffinity-based, and dynamic techniques, with nanofilters, nanoarrays, and nanoporous membranes representing typical size-based platforms [48]. Commercially available EV isolation kits offer rapid and simplified workflows; however, variability in specificity, reliability, and cost-effectiveness remains a concern. Despite their convenience, these kits remain expensive and are generally restricted to low sample processing capacity [32]. A schematic overview illustrating the major techniques commonly employed for EV separation and purification is presented in Figure 2.

Microfluidic chip-based platforms enable the integrated isolation and detection of EVs with reduced sample volume, high throughput, and compatibility with downstream analysis. Immunoaffinity-based microfluidic isolation (Mf-IAC) selectively captures EVs via known surface antigens and has proven effective for isolating EVs from culture media and body fluids. Nevertheless, its reliance on pre-established markers restricts capture to particular subpopulations, which might be suitable for therapeutic applications but less ideal for EV-based diagnostics where broad EV profiling is required [49]. Consequently, antigen-independent, size-based microfluidic filtration (Mf-F) approaches have gained prominence, enabling unbiased EV capture based on physical properties. Early Mf-F devices used nanoporous membranes in pressure- or electrophoresis-driven configurations to separate EVs from cells and debris, with electrophoresis overcoming pore clogging and improving purity [50]. Modular sequential nanoporous microfluidic systems enhance EV isolation by improving throughput and purity and reducing protein carryover relative to ultracentrifugation [51]. Similarly, modular platforms such as ExoTIC enable high-yield, size-selective EV isolation with lower protein contamination [52].

## 6. Characterization of EVs

Size, concentration, morphology, presence of cargo (e.g., protein markers, DNA, RNA), protein concentration, and other characteristics are frequently used to characterize EVs after they have been isolated [32]. Physical characteristics, molecular makeup, or cell of origin can all be used to characterize purified EVs. Electron microscopy, dynamic light scattering (DLS), and nanoparticle tracking analysis (NTA) are commonly employed to determine the size and concentration of EVs. Proteomic analysis, immunoblotting, or flow cytometry are commonly used for biochemical studies of EVs [53].

## 7. Cargo of EVs

EV function in the TME is determined by its cargo as well as the dynamics of its secretion and internalization. The cargo of EVs—including membrane receptors, proteins, nucleic acids, and lipids—reflects the molecular composition of the originating cells and facilitates the transfer of signals between cells [54]. It has been discovered that mRNAs and miRNAs are important parts of EVs. More RNA species, including as long non-coding RNAs (lncRNAs), transfer RNAs (tRNAs), and viral RNAs, can now be seen thanks to advancements in EV detection methods [55]. Additionally, various RNAs, including lncRNA, play important roles in influencing cancer cell growth [56].

According to recent research, proteins interact with elements of the EV biogenesis machinery to become integrated into EVs. In a similar manner, tetraspanins play a key role in directing membrane proteins into EVs, either by direct association or by trapping them in tetraspanin-enriched microdomains. As tumor exosomes transport growth factors, immunomodulators, and oncoproteins, understanding the protein–protein interaction networks responsible for cargo selection could reveal new approaches to suppress EV-driven oncogenic signaling [57].

RNAs are found in mammalian EVs, as well as in bacterial, fungal, insect, parasitic, and plant EVs. This suggests that this messaging mechanism is a conserved function. Indeed, additional research has revealed that the loaded RNA precisely mediates the functional effects of EVs on recipient cell populations’ cellular proliferation and viability, suggesting a broad role for EV-loaded RNA in both healthy and pathological contexts [58]. The capacity of EV-carried mRNA molecules to be translated into proteins shows that genetic material can move horizontally between cells without the need of viruses. In addition to mRNAs, exosomes are highly enriched in short non-coding RNAs, highlighting the significant role of gene regulation in their function. Exosomes carry short RNAs that can control the gene expression of recipient dendritic cells. However, there is evidence linking several EV-associated miRNAs to malignancy [57].

A previous study unequivocally shows that EVs include a far wider variety of RNA species. Furthermore, identifying which EV subpopulations contain specific RNAs and how this relates to EV biogenesis is crucial to comprehending the physiological roles of EVs because the RNA species found in EVs are probably as diverse as EVs themselves [58].

Fragmented mitochondrial DNA (mtDNA), genomic DNA (gDNA), and even parasite DNA have been found in EVs. EV-associated DNA mostly resides on the vesicle surface and is often packaged in nucleosome-like structures through association with histone proteins. Although DNase treatment is routinely applied to remove free DNA from EV preparations, surface-associated DNA may retain partial protection due to protein binding. According to recent research, genomic DNA fragments can be transported to the cytoplasm after DNA damage, where it engages cytoplasmic DNA sensing pathways and cause apopotosis or cellular senescence. Remarkably, DNA fragments secreted by EVs help maintain cellular homeostasis by preventing cytoplasmic DNA sensors from activating [57]. In a study by Fernando et al., comparative analysis of DNA isolated from whole plasma and plasma-derived exosomes demonstrated that more than 90% of circulating cell-free DNA was associated with exosomes, in contrast to earlier reports that primarily attributed circulating DNA to larger extracellular vesicle populations [59].

When early endosomes develop into late endosomes and acquire ILVs, their biogenesis is one of the possible processes by which DNA is loaded into exosomes. Although the relationship between cytosolic DNA levels and EV-DNA has not yet been proven, the sequestration of proteins, lipids, and cytosol during the creation of ILVs may result in the encapsulation of cytosolic DNA within the vesicles. Evidence suggests that instability of the micronuclear envelope can lead to micronuclear collapse and the release of nuclear content into the cytoplasm, where gDNA may be trafficked to MVBs and ultimately packaged into exosomes [60].

## 8. Effects of EVs in TME

Because of their function in tumor immunomodulation, EVs have garnered a lot of attention lately. EVs contribute to both tumor initiation and the modulation of immune evasion mechanisms. They enable cell-to-cell communication via the transfer of nucleic acids, proteins, and other biologically active molecules. In particular, by disrupting immune cell function or altering immunosuppressive pathways, EVs might help tumor cells avoid immune surveillance and attack, which will accelerate tumor growth and metastasis. However, they may convey immunomodulatory substances that promote immune system activation and control, strengthening the body’s defenses against cancer. EVs’ dual purpose offers tumor immunotherapy potential targets and pathways [16]. Table 1 compiles representative studies that elucidate the mechanistic roles of EVs.

### 8.1. Role of EVs in Angiogenesis

Angiogenesis refers to the establishment of new blood vessels from existing vasculature and is crucial for development, tissue repair, and various disease processes. This process creates and transforms a circulatory system into a sophisticated vessel system that mediates numerous essential physiological functions, such as temperature regulation, waste removal, immunological response, tissue oxygenation, nutrient delivery, and blood pressure maintenance. Depending on their cellular origin, EVs use a variety of ways to target the critical phases of vessel development and actively affect the process of blood vessel formation. VEGF is a notable angiogenesis mediator among the functional proteins present in EVs. Although various functional proteins and miRNAs related in angiogenesis are included in the complexity of the EV content, more investigation is required to clarify their precise functions in angiogenesis [77].

**Figure 3:**
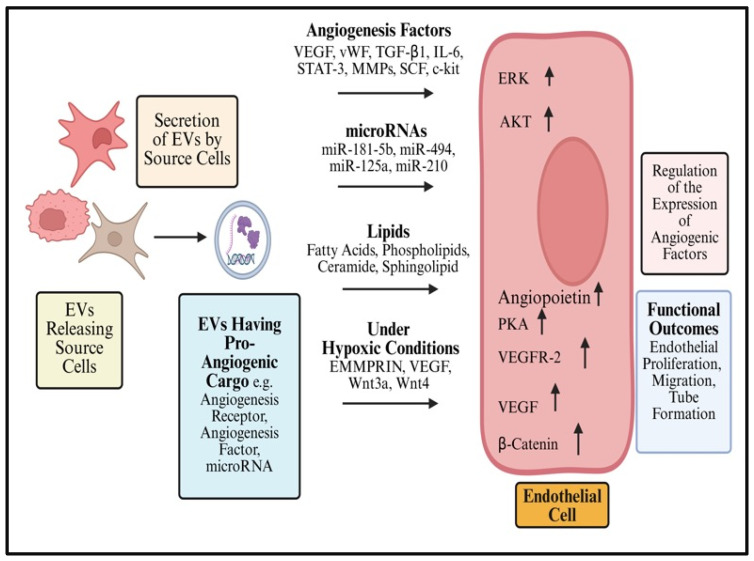
EV-mediated paracrine activity of mesenchymal stem cells (MSCs) in angiogenesis. MSCs release EVs enriched with bioactive lipids, microRNAs (miRNAs), and pro-angiogenic molecules, including growth factors, chemokines, and cytokines. Upon transfer to recipient endothelial cells, MSC-derived EV cargo activates pro-angiogenic signaling pathways that are essential for endothelial cell proliferation, migration, and vascular remodeling during tissue repair. Under hypoxic conditions, MSCs secrete EVs with enhanced angiogenic potency, which further modulate endothelial signaling pathways and regulate the expression of angiogenic factors, thereby promoting neovascularization (created in https://BioRender.com).

By modulating angiogenic processes, EVs derived from mesenchymal stem/stromal cells (MSCs) facilitate tissue repair in ischemic injuries. MSCs-EVs restore blood flow by modifying the activities of cells, including progenitors, stem cells, and endogenous mature cells [78]. The mesenchymal stem cells are encouraged by hypoxia to generate EVs containing proteins associated with the NK-kB pathway and active pSTAT3. When these EVs reach the EC, they promote the synthesis of proangio-genic proteins, platelet-derived growth factor (PDGF), epidermal growth factor (EGF), fibroblast growth factor (FGF), stem cell factor (SCF), vascular endothelial growth factor (VEGF), and KIT proto-oncogene receptor tyrosine kinase (c-kit) are among the growth factors that are present in EVs and are transported to the EC. The wnt present in EVs stimulates the transcription of molecules essential for angiogenesis when it interacts with its receptors. By transporting different microRNAs, EVs aid in angiogenesis (Figure 3). Interestingly, miR-125 improves tip cell specification by lowering DLL4, while miR-31 blocks an inhibitor of HIF-1-α, allowing HIF-1-α activation and the subsequent expression of VEGF and PDGF, thereby stimulating (neo)angiogenesis [77].

### 8.2. Effect of EVs on Fibroblasts

Cancer-associated fibroblasts (CAFs) are activated stromal cells within the TME that promote tumor growth, angiogenesis, metastasis by secreting growth factors, MMPs, chemokines, and ECM components [79]. Different molecular pathways in the communication between cancer cells and the stromal components of the TME are impacted by EVs. TME cells can exhibit pro-tumorigenic activities, such as inducing CAF, pro-inflammatory, and pro-angiogenic phenotypes, through the influence of various cargos found in EVs released by cancer cells. The composition of stromal cell-produced EVs can change when a CAF phenotype is activated in those cells. It has been demonstrated that CAF-derived EVs accelerate the growth of tumors by encouraging the development of more aggressive traits in cancer cells, such as enhanced growth, migration, invasion, metastasis, and resistance to treatment. Furthermore, there is proof that EVs generated from CAFs and cancer cells might affect the microenvironments at remote locations and encourage the development of pre-metastatic niches [80].

### 8.3. Effects of EVs on Endothelial Cells

Tumor extravasation involves the stepwise adhesion of circulating tumor cells to ECs through selectins and integrins, followed by transendothelial migration via intercellular junctions (diapedesis). During this process, tumor cells actively induce endothelial contraction, creating junction gaps that facilitate invasion into the surrounding extracellular matrix while simultaneously triggering proinflammatory signals. Cancer cells orchestrate the behavior of vascular-associated cells, reshaping the local microenvironment. Microthrombus formation and platelet aggregation further increase inflammation and activation of microvascular ECs, thereby promoting the establishment of a metastatic vascular niche [81].

Transferring functional cargos including proteins, cirRNAs, lncRNAs, and miRNAs can result in EV-mediated signaling in endothelial cells. Furthermore, endothelial cells can trigger receptor-mediated signaling in response to membrane-bound proteins in EVs [82]. EVs interact with endothelial cells by various mechanisms, including direct activation of cell surface receptors, endocytosis, and membrane fusion. Vascular endothelial growth-factor receptor-2/3 and members of the Notch pathway are among the important signaling proteins and membrane receptors that are regulated by the endothelial-vesicular network, an emerging mechanism that is crucial in forming the tumor vasculature [83]. Although it is yet unclear what molecular mechanisms underlie EVs’ role in vascular homeostasis and disease in humans, they influence endothelial cell communication in the microenvironment [84].

### 8.4. EVs in Epithelial to Mesenchymal Transition

**Figure 4:**
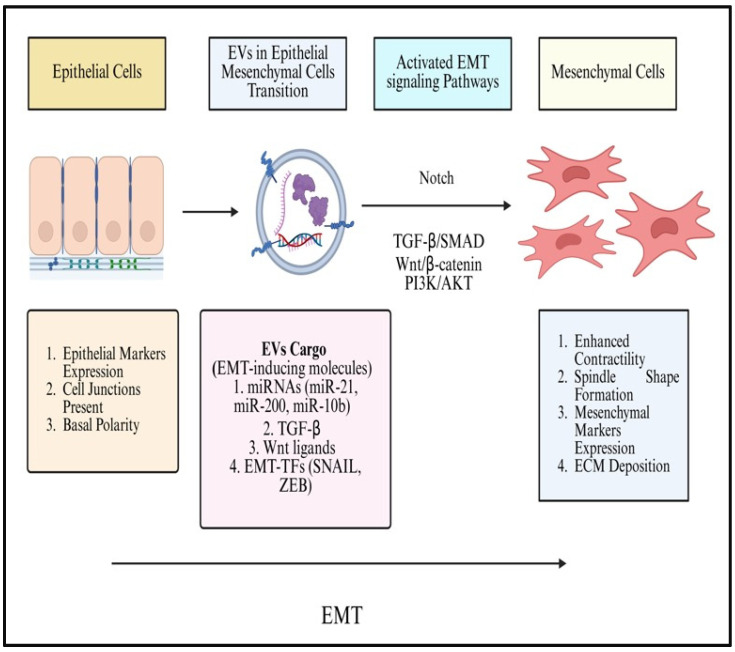
EVs in epithelial–mesenchymal transition. Extracellular vesicles (EVs) released from epithelial cells transfer EMT-inducing cargo, including miRNAs, growth factors, and transcriptional regulators, to recipient cells, activating TGF-β/SMAD, Wnt/β-catenin, PI3K/AKT, and Notch signaling pathways, thereby promoting epithelial–mesenchymal transition and mesenchymal phenotypic conversion (created in https://BioRender.com).

Epithelial–mesenchymal transition (EMT) is a key process in embryonic development and also contributes to certain pathological conditions such as tumor progression and fibrosis. The EMT process involves several molecules, factors, mediators, and signaling pathways [85]. EMT represents a conserved cellular reprogramming process in which epithelial cells lose polarity and cell–cell adhesion while acquiring mesenchymal traits. Apart from its pivotal function in development, EMT has been linked to a cellular mechanism during carcinogenesis that promotes the invasion and metastasis of tumor cells. The induction of mesenchymal-associated genes by specific signaling cascades and transcription factors is a key determinant of EMT. Secreted proteins and peptides as well as exosomes, are examples of secretome components that have been identified as having an impact on EMT [86]. According to new research, T-EVs, such as exosomes and microvesicles, actively stimulate the EMT (Figure 4) in cancer and stromal cells by delivering oncogenic cargoes that cause recipient cells to change into more invasive, mesenchymal phenotypes and accelerate the spread of metastases [87]. The overexpression of HRAS in Madin–Darby canine kidney epithelial cells promotes the incorporation of mesenchymal markers, such as vimentin and MMPs into exosomes, which can induce EMT in recipient cells [88].

### 8.5. Role of EVs in the Creation of New Pre-Metastatic Niche

The pre-metastatic niche (PMN) refers to a supportive microenvironment at distant sites that facilitate the survival and growth of incoming circulating tumor cells. Stephen Paget’s 1889 observation that certain tumor forms have a tendency to spread to different organs, indicating that the microenvironment influences metastatic invasion, served as the basis for the PMN idea [87,89]. EVs may contribute to PMN production and metastatic dissemination through numerous mechanisms. For example, exposure of breast cancer cells to the chemotherapeutic drug Paclitaxel enhances the secretion of T-EVs. These T-EVs travel through the blood system to the lungs, where they increase fibronectin and alter the extracellular matrix to promote tissue permeability. By making the lung tissue less rigid, the ECM alteration promotes an environment that helps cells develop a PMN, which facilitates the spread of metastatic disease [90,91].

Moreover, maintenance of cholesterol homeostasis has been shown to facilitate EV-mediated signaling from prostate cancer cells, thereby promoting PMN formation and metastatic dissemination to the bone marrow. According to some theories, T-EVs can enhance osteoclast differentiation by upregulating the NF-kB pathway in response to elevated cholesterol production. PMN development and the advancement of the bone’s metastatic process are made possible by this procedure [92]. Breast cancer-derived microvesicles deliver miR-122 to stromal cells, where it suppresses pyruvate kinase, inhibits glucose uptake, and promotes PMN formation by increasing glucose availability for tumor cells [72]. Imaging of T-EVs in metastatic organs demonstrates dynamic EV–target cell interactions that promote stromal cells reprogramming at post-metastasis sites within PMNs [93,94]. Tumor-secreted exosomes facilitate the recruitment of diverse cell populations, including fibroblasts, macrophages, endothelial cells, and specific subsets of bone marrow-derived cells (BMDCs) to the PMN [69,71].

### 8.6. EVs in Metastasis

The process of metastasis involves several steps and involves different cell types in different organs. EVs have recently been recognized as major regulators of short- and long-range intercellular communication among diverse cell types during multiple stages of metastasis, complementing the well-established functions of tumor- and host-derived cytokines. By modulating immune, vascular, and stromal compartments, EVs contribute substantially to the complex tumor–host interplay. Through these mechanisms, EVs regulate key metastatic processes such as tumor plasticity and heterogeneity, vascular remodeling, immune modulation, cancer-niche coevaluation, and the establishment of PMN environments [95].

EVs influence many stages of cancer metastasis, including conditioning the PMN, influencing immune systems, and stimulating angiogenesis. Cancer-derived EVs educate primary and distant tumor environmental cells, and this helps in the spread of cancer. Cancer-derived EVs lower the host immune system, stimulate angiogenesis or break tight junctions in ECs, cause the transformation of cancer-associated fibroblasts (CAFs), and establish a premetastatic niche during the metastatic phase. Furthermore, EVs derived from cancer-associated cells, like MSCs and CAFs, can facilitate cancer spread [96].

### 8.7. EVs and Immunomodulation

As tumors develop, extracellular vesicles have become important immune response regulators. A wide variety of molecular cargo found in EVs is essential for immunomodulation. B cell-released EVs triggered T cell immunological responses in addition to carrying major histocompatibility complex (MHC) class II molecules. The dendritic cell (DC) tiny EVs cause tumor suppression in vivo. DC tiny EVs indirectly stimulate T cells. In a highly interconnected network of immunological responses, EVs generated by immune cells continuously balance the autoimmune and immune regulatory responses of neighboring cells [97]. The patient’s innate and adaptive immune systems usually fail to identify and eliminate a tumor. In fact, cancer cells actively remodel the tumor milieu to suppress immune surveillance. One of the best-characterized mechanisms of tumor-induced immunosuppression is the expression of programmed death-ligand 1 (PD-L1) on the surface of cancer cells. The cell surface receptor known as programmed death 1 (PD-1) is liganded by PD-L1. PD-L1 on the surface of cancer cells interacts with PD-1 expressed by immune cells during TME surveillance, resulting in the activation of immune checkpoint pathways. Signaling processes that prevent immune cell development and function are facilitated by PD-1 activation [98].

Remarkably, it has also been documented that EV-associated PD-L1 directly inhibits T cell activity. EVs are important regulators of the immune response against cancer, with DC-derived EVs playing a key role in triggering antitumor immune responses. In contrast, EVs may influence T-cell activity through both direct interactions and indirect mechanisms involving the inhibition of dendritic cell function. Notably, T-EVs can impair antitumor immunity by promoting the induction, differentiation, and expansion of regulatory T cells, thereby contributing to immune suppression [99].

### 8.8. Role of EVs in ECM Remodeling

By altering the components of macromolecules, degradation enzymes, and stiffness, the extracellular matrix (ECM) contributes significantly to the development of tumors. Through the abnormal activation of signaling pathways, the interaction of ECM components with numerous surface receptors, and mechanical impact, cellular components in the tumor tissue control these changes. Furthermore, the cancer-shaped extracellular matrix controls immune cells, creating an immunological-suppressive milieu that reduces the effectiveness of immunotherapies. As a result, the ECM promotes tumor growth and serves as a barrier to shield cancer from therapies. The design of customized anticancer treatment is hampered by the extensive regulatory network of the ECM remodeling [100].

The influence of T-EVs causes the stromal cells’ molecular and cellular surroundings, as well as their extracellular proteins and enzymes, to change dynamically during the growth of malignant tumors. It is widely accepted that ECM remodeling makes tumors more invasive. The ECM substance fibronectin is carried by T-EVs, which promotes cellular motility and aids in the formation of early adhesions [101].

Proteomic study of T-EVs demonstrated that annexins, ADAM10, and α-3 integrin were abundant in T-EVs and linked to cell movement and regional invasion [102]. ARF6, Cav-1, MMP9, and MMP2 are among the several bioactive chemicals found in large T-EVs that are linked to regional invasion; their abundance is also linked to the formation of tumors [103]. Invadopodia are EV docking sites that promote cell invasion by accelerating ECM destruction by localized secretion of metalloproteinase MT-1-MMP [104].

## 9. EVs as Biomarkers of Metastatic Diseases

A biomarker is an objectively quantifiable indicator that reflects normal physiological states, pathogenic alterations, or treatment responses. EVs are documented to be attractive, less invasive biomarkers for cancer immunotherapy, providing critical information on immune regulation within the TME. EVs are key contributors to drug resistance and exhibit distinct qualitative and quantitative features under both physiological and pathological conditions such as cancer [105].

Because the bioactive cargo of EVs mirrors the physiological or pathological state of their parent cells, EVs are considered highly valuable biomarkers for disease diagnosis and prognosis [106]. While investigating and identifying EV cargo will undoubtedly help improve diagnosis and prognosis, it is crucial to remember that cancer cells release more EVs, and the amount of EVs in the blood rises as the disease progresses, reaching larger levels in advanced stages. Furthermore, it is known that T-EVs are present in every bodily fluid. This means that the changed molecules present in tumor cells can be evaluated in the circulation, urine, tears, cerebrospinal fluid, and other fluids, even if they are limited to the primary lesion [107]. Several studies have demonstrated the promise of EVs in cancer liquid biopsy. These include the identification of wild-type EGFR amplification and EGFRvIII mutations in EVs isolated from the cerebrospinal fluid of glioblastoma patients, EGFRvIII mRNA in plasma-derived EVs from glioma patients, and tumor-associated transcripts in circulating EVs from prostate cancer patients. Therefore, EVs offer a promising avenue for more precise disease diagnosis and prognostic assessment [107].

**Figure 5:**
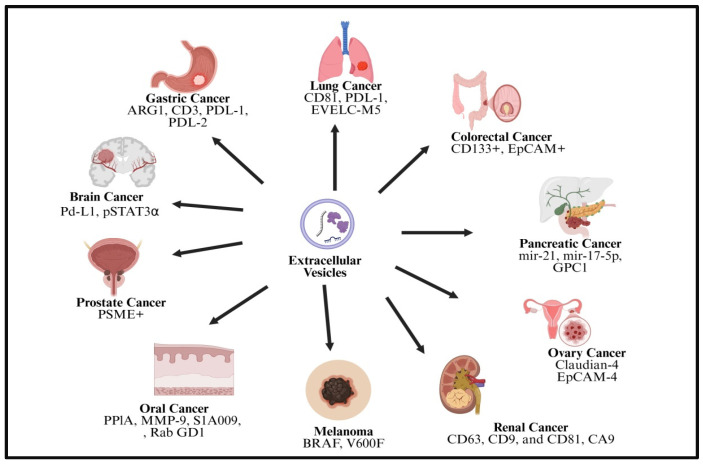
Clinical diagnostic/prognostic utility of extracellular vesicles with organ specific cargo (created in https://BioRender.com).

Because of their inherited qualities, EVs are excellent options for monitoring therapy response and assisting in the diagnosis and prognosis of cancer due to the presence of various cargos (Figure 5). One of their benefits as a non-invasive testing technique is their capacity to circulate through different physiological fluids. Furthermore, the lipid bilayer of EVs provides protection for proteins and RNA against enzymes, enabling reliable genomic testing. The functional relevance of EVs in early cancer detection and monitoring is highlighted by their elevated levels in patients and the distinct proteomic and nucleic acid cargo profiles compared to normal cells [105].

EVs are thought to be potential biomarkers for both tumor growth and metastasis in colorectal cancer, and patients with CRC have considerably greater levels of EV-associated mir-17a-5p, particularly those with distant metastases and higher clinical stages [108]. Elevated serum levels of miR-934, transported by tumor-derived exosomes (EXOs), have been associated with CRC and liver metastasis, suggesting their potential utility as biomarkers. miR-934 can cause M2 macrophages to polarize, which in turn downregulates PTEN expression and activates the PI3K/AKT pathway. Therefore, it might encourage the development of metastatic niches and liver metastases through the release of the chemokine CXCL13, which would activate the CXCL13/CXCR5/NFκB/p65 pathway and create an inflammatory milieu [109].

Elevated miR-21 expression in serum EVs suggests their usefulness in differentiating metastatic from non-metastatic CRCs [110]. Additionally, miR-21 is linked to breast cancer metastases; individuals with bone metastases from this form of cancer had greater levels of miR-21. Since miR-21 seems to affect osteoclast formation and function via controlling PDCD4 expression, it may be a valuable biomarker for identifying bone metastases associated with breast cancer [111].

In non-small cell lung cancer (NSCLC), proteomic profiling has revealed that specific liposaccharide-binding proteins present in serum-derived exosomes differ between individuals with or without metastases, suggesting their potential utility as biomarkers alongside miRNAs [112]. According to in vitro research, EVs released by hepatocytes infected with replicating HBV cause monocytes to produce programmed cell death 1 ligand 1 (PD-L1), which may inhibit host antiviral action [113]. Interestingly, Montaldo and coworkers reported that plasma-derived EVs from HCV patients treated with direct-acting antivirals exhibited reduced levels of miR204-5p, miR143-3p, miR181a-5p, and miR-122-p compared with healthy individuals [114]. EVs secreted by various TME cells may contribute to the beginning and progression of RCC. Jiang and colleagues reported that EVs derived from RCC cell line OS-RC-2 promote tumor cell proliferation through a p-AKT-dependent manner and reduce the expression of hepaCAM, a tumor suppressor commonly lost in a variety of human cancer types [115]. Du et al. demonstrated that human Wharton’s jelly mesenchymal stem cell-derived EVs enhance RCC cell proliferation and aggressiveness by stimulating hepatocyte growth factor (HGF) production and activating AKT and ERK1/2 signaling pathways, as shown in both in vitro models and nude mouse xenografts [116].

Circulating EV-associated integrin β1 has emerged as a promising biomarker for predicting salivary adenoid cystic carcinoma (SACC) metastasis, which most frequently spreads to the lungs. Notably, integrin α2β1 carried by CAF-derived EVs is detectable at high levels in the plasma and has been shown to reprogram lung fibroblasts toward a pro-tumorigenic condition, thereby facilitating the establishment of a pulmonary PMN [79]. Alterations in circulating prostate cancer-derived EVs have been related to disease severity and recurrence risk, indicating their potential as prognostic biomarkers [117].

EVs have many benefits over circulating tumor DNA (ctDNA), despite ctDNA being another essential element in cancer diagnosis and prognostics. The first is that compared to ctDNA, there are more mutant copies accessible for sampling since the EVs are made of RNA. Unlike ctDNA, which is passively evacuated from necrotic or apoptotic cells, mRNAs in EVs are actively released. Because of their consistent size, these EVs are simple to identify using electron microscopy. Additionally, the lipid bilayer shields EV cargos from deterioration, making them acceptable for research [118]. Table 2 summarizes the diagnostic and prognostic potential of EVs.

## 10. Therapeutic Applications of EVs in Cancer Management

**Figure 6:**
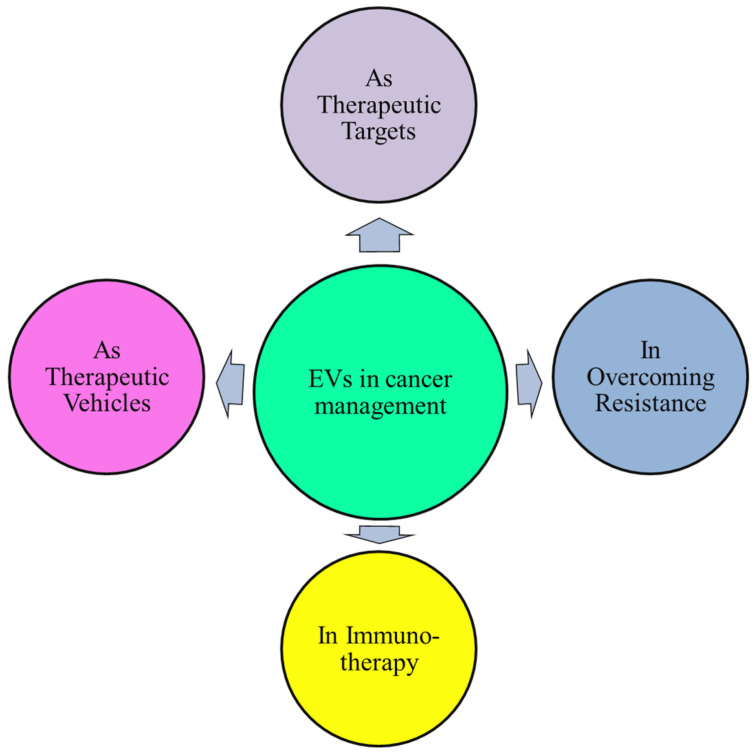
Therapeutic implications of EVs in cancer therapy. This figure provides a schematic representation of the multifaceted roles of extracellular vesicles (EVs) in cancer management, highlighting their applications as therapeutic targets, therapeutic vehicles, modulators of immunotherapy, and contributors to overcoming therapy resistance.

Because EVs inherit characteristics from their parent cells, they can be used to diagnose and treat diseases. First, EVs are showing promise as a liquid biopsy tool. Certain molecules found in EVs released by diseased cells in bodily fluids may indicate the beginning and course of the disease [137]. According to reports, EVs are potential therapeutic agents for the management of solid tumors, both as supplements that improve the efficacy of well-established therapeutic approaches and as stand-alone treatments (Figure 6). EVs can be employed as therapeutic delivery vehicles, either in their natural form or as engineered vesicles, to transport bioactive compounds and pharmacological agents [138].

### 10.1. EVs as Therapeutic Vehicles to Deliver Bioactive Molecules

The therapeutic promise of EVs in oncology is largely attributed to their ability to efficiently transport a wide range of bioactive cargos. Because EVs are neither transformational nor replicative, they cause fewer negative side effects. Furthermore, the targeting techniques, administration route, and cell source all affect how EVs are biodistributed [139]. Because of their many biological characteristics, using natural or genetically-engineered EVs as medication delivery vehicles has unique benefits. Researchers are attempting to use exosomes as nanoparticle-based delivery systems for the treatment of cancer since they are thought to be a natural means of delivering bioactive chemicals with minimal cytotoxicity [140].

EVs have shown to transport proteins and nucleic acids across plasma membrane barriers with minimal cytotoxic effects. Researchers in one study discovered that EVs are more successful than liposomal delivery systems when comparing their biological effects. Consistent with this, exosomes have been used as delivery vehicles for small interfering RNA targeting Kras^G12D in mouse models of pancreatic cancer, showing superior efficacy compared with liposome-based administration [141]. To more effectively treat brain cancers, a variety of compounds, including therapeutic agents, small interfering RNAs (siRNAs) and miRNAs, can be integrated into exosomes and transported across the blood–brain barrier [142]. In rat models, exosomes derived from bone marrow stromal cells have been used to deliver miR-146b, resulting in the suppression of malignant glioma development [143].

### 10.2. EVs as Therapeutic Targets for Cancer Treatment

EVs have been implicated in multiple processes that promote metastasis. Thus, in order to decrease metastasis, EV-based cancer treatment strategies have concentrated on preventing T-EV release, transit during the journey, and absorption. Cytokines, chemokines, or growth factors are the primary means by which cells in the TME exchange information with one another. However, EVs produced from the TME’s cells also mediate communication within the TME [144]. In fact, EVs are crucial for the growth of tumors. Consequently, EV generation increases dramatically during TME maturation, accelerating tumor development. It should come as no surprise that lowering the quantity of T-EVs could offer a treatment option to stop the spread of malignancy. Three different EV-targeting treatment approaches have been put forth thus far: removing circulating EVs, suppressing EV secretion, and interfering with EV absorption [145]. These strategies, whether applied independently or alongside established treatments, represent a promising and potentially transformative avenue in cancer therapy.

Extensive in vitro, preclinical in vivo, and clinical investigations have demonstrated that a variety of compounds can suppress the biogenesis and release of exosomes and other EVs. At various phases of exosome formation and release, these substances target distinct proteins. Many exosome inhibitors target other proteins at various phases of exosome formation and release [146]. Furthermore, as research on exosome biology advances, the significance of using exosome inhibitors as supplemental drugs for cancer treatment will progressively grow. As a result, new strategies that target exosomes to reduce the pathological communication linked to cancer are probably going to have a big therapeutic impact on cancer patients in the future. Reducing exosome release by treating small cell lung cancer (SCLC) cells with GW4869 and Nexinhib20 dramatically triggered apoptosis and successfully decreased cellular growth [147]. Although evidence remains limited, in vitro studies and a small number of preclinical in vivo investigations suggest that several compounds may interfere with exosome and microvesicle biogenesis and release. Bisyndoylmaleimide I, manumycin A, U0126, GW4869, clopidogrel, NSC23766, imatinib, dimethyl amiloride, indomethacin, chloramidine, cytochalasin D, calpeptin, Y27632, glibenclamide, pantethine, sulfisoxazole, and imipramine are among them [148]. Novel approaches, such as genetic engineering and pharmaceutical inhibitors, are used to specifically suppress tumor-derived exosomes [149].

### 10.3. EVs in Immunotherapy

EVs are increasingly being recognized as a viable cell-free immunotherapeutic platform, showing great promise in preclinical and clinical research. These vesicles, including microvesicles and exosomes, can modulate immune responses within the tumor microenvironment, making EVs attractive tools for cancer immunotherapy, either as vaccine platforms or as delivery vehicles for targeted therapeutic agents. Incorporation of immunomodulatory compounds or gene-editing tools (GETs) into EVs has shown encouraging results in enhancing anti-tumor immune responses and counteracting the immunosuppressive TME. Furthermore, enhancing the clinical effectiveness of EV-based treatments requires refining delivery systems using both passive and active targeting techniques. Either the direct transformation of natural EVs or parent cell engineering can produce engineered EVs that target the TME [150].

Under physiological conditions, exosomes are implicated in the presentation of antigen to T cells, the development of effector functions, the priming of T cells, the maturation of immune cells, and the activation of immune responses through pathways restricted by the major histocompatibility complex (MHC). Research indicates that EVs directly contribute to tumor immunological invasion via a number of pathways, including inducing T cell death. T-EVs expressing immune-related ligands such as Fas-ligand (CD95L) on their surface have been frequently reported to induce apoptosis in T cells upon direct interaction with ligand-positive cells [151].

SMART-Exos are genetically modified EVs engineered to express two different monoclonal antibodies, enabling the concurrent engagement of T cells and cancer cells; studies have shown that this dual targeting strategy significantly enhances anticancer activity [152,153]. Shi et al. developed αCD3–αHER2 SMART-Exos by genetically engineering exosomes to express antibodies against human CD3 and HER2, enabling simultaneous targeting of T cells and HER2-positive breast cancer cells [152]. SMART-Exos expressing dual monoclonal antibodies against CD3 and EGFR have been reported to simultaneously bind T cells and EGFR-positive triple-negative breast cancer (TNBC) cells [153]. The results from a previous study point to exosomes as having strong therapeutic potential in immunotherapy for cancer. Multiple clinical trials are currently underway to assess the efficacy of EVs in immunotherapy, alone or combined with other anticancer treatments, to enable the clinical adoption of EV-based therapeutic approaches [154].

### 10.4. EVs in Overcoming Resistance

In cancer treatment, drug resistance is a significant problem that frequently results in treatment failure and disease recurrence. Even with improvements in targeted therapy and chemotherapeutic medicines, malignancies frequently become resistant to these drugs, rendering them useless. The endogenous origin, biocompatibility, and barrier-crossing capabilities of EVs make them promising platforms for therapeutic delivery. It may be possible to reduce systemic toxicity, increase drug accumulation at target areas, and target certain cells with precision by utilizing the special qualities of EVs [155].

In glioblastoma (GBM) cells resistant to temozolomide (TMZ), miR-9 is increased and contributes to the production of the drug efflux transporter P-gp. To reduce the expression of proteins associated with drug resistance, Munoz et al. encapsulated anti-miR-9 within EVs derived from MSCs [156]. Lipidomimetic chain-conjugated HA (lipHA) was introduced into EV membranes by Liu et al. Doxorubicin resistance was overcome by incorporating the drug into lipHA-hEVs, which effectively lowered drug efflux by downregulating P-gp expression in resistant MCF7/ADR cells [157].

Cisplatin packaged in EVs derived from M1 and M2 macrophages accumulates in tumors and markedly enhances cytotoxicity against resistant A2780/DDP (cisplatin-resistance cell derivative of human ovarian cancer cell lines) and A2780 cells (human ovarian cancer cell lines) [158]. One common clinical treatment for metastatic peritoneal cancer is hyperthermic intraperitoneal chemotherapy, or HIPEC. Low drug penetration efficiency and the quick emergence of resistance, however, restrict its effectiveness. Genetically modified exosome-thermosensitive liposome hybrid nanoparticles (gETL NPs) were created by Lv et al. to improve drug delivery effectiveness and combat drug resistance [159].

## 11. EVs as Vaccine

A promising treatment strategy to improve specific T-cell immunity against the majority of solid tumors is the use of cancer vaccines. These vaccines target tumor cells and work well in conjunction with immune checkpoint inhibition or other immunotherapies to promote anti-tumor immunity, eliminate minimal residual disease, and reduce side effects. Nevertheless, limited immunogenicity, pronounced tumor heterogeneity, an immunosuppressive TME, and suboptimal delivery methods continue to pose major challenges to the development of tumor cell-based vaccines. EVs, on the other hand, are thought to be the best medication carriers and vaccination platforms since they are spontaneously released by cells. EVs exhibit improved tissue delivery capabilities, provide highly organ-specific targeting, and elicit more extensive and efficient immune responses. The advancement of cancer immunotherapy depends on the creation of EV vaccines. EV vaccines made using Good Manufacturing Practices (GMP) have several benefits over cell-based vaccinations, including excellent safety, convenience of storage and transportation, and a variety of sources [160].

EVs are renowned for being a plentiful supply of immune molecules and antigens that can be utilized to build vaccines for both humans and animals. EV-based vaccination has the potential to greatly boost immune responses against a variety of diseases, including SARS-CoV-2, lymphocytic choriomeningitis virus (LCMV), Marek’s disease virus (MDV), and porcine reproductive and respiratory syndrome virus (PRRSV). The created and modified EVs demonstrated a remarkable potential in the creation of anti-tumor vaccines and therapies, protection against parasitic diseases (e.g., Eimeria and Plasmodium yoelii) and viral infections (e.g., COVID-19), and improvement of biomarkers. Also, EVs possess a key role in antigen presentation in vivo [161].

In order to change their composition, synthetic EVs have been purposefully altered either after secretion or by genetically modifying the secreting cells. Innovative technologies, such as light controlled protein loading and high-density EV platforms, allow for the precious incorporation of immunogenic proteins into EV membranes or lumens, creating synthetic EVs for targeted vaccination [162]. Collectively, these findings highlight the potential of extracellular vesicles-based combined mRNA and protein vaccine platform (EVX-M+P) as a feasible platform for the simultaneous delivery of mRNA and protein antigens for the prevention of cancer and infectious diseases. EVX-M+P represents an EV-based vaccination strategy that integrates both mRNA and protein components [163].

Using BL6 melanoma cells, exosomes from ovalbumin-pulsed dendritic cells (OV-Pulsed DCs) and their absorption by CD4+ T cells promoted the growth and development of central memory cytotoxic T lymphocytes (CTLs) and prevented Treg suppression in vitro. Additionally, three months following immunization, C57BL/6 mice given OVA-pulsed dendritic cell-derived exosomes (OVA-pulsed DEXs) exhibited a higher quantity of OVA-specific CD8+ CD44+ T cells than the control group [164]. Priming particular T cells via DEXs is for DCs to first capture and digest the exosomes is a significant effect. DEXs from mature DCs are more effective at stimulating T cells than DEXs from immature DCs, according to a study that used direct DEX-T cell interaction [165].

As mRNA and protein medication carriers, earlier researchers produced room-temperature stable inhaled lung-derived EVs, or exosomes (lung-Exos). In a vaccination study, lung-Exos loaded with mRNA encoding SARS-CoV-2 spike (S) protein elicited stronger immunoglobulin G (IgG) and secretory IgA (SIgA) responses compared with spike mRNA-loaded liposomes (S-Lipo). According to the findings, EVs may be a better inhaled mRNA medication delivery method than manufactured liposomes [166].

Innate-like T cells with two anticancer functions are called γδ-T cells. The dual anticancer capabilities of γδ-T calls are retained in γδ-T-derived EVs, or γδ-T-EVs. These γδ-T-EVs exert immunoadjuvant effects on antigen-presenting cells, as demonstrated by the enhanced expression of co-stimulatory and antigen-presenting molecules, increased secretion of pro-inflammatory cytokines, and improved antigen-presenting capacity of DCs following γδ-T-EV exposure. Based on these properties, tumor-associated antigens (TAAs) were incorporated into γδ-T-EVs to generate an EV-based vaccine, which stimulated stronger tumor-specific T-cell responses than uploaded γδ-T-EVs. The vaccination regimen also caused tumor cell death and maintained direct anticancer effects [167].

Helminth EVs might influence IL-33-mediated signaling as well as alternative activation, establishing them as essential parasite virulence factors. Additionally, immunization that targeted EVs elicited protective immunity against the parasite, emphasizing their crucial promise in the initiation of infection [168]. EV-based vaccinations have several advantages over conventional ones, but key problems still need to be resolved before they can be quickly used in clinical settings. Specifically, the selection and validation processes for the best antigen-adjuvant combination take a lot of time. Moreover, this intervention should only include antigens that are strongly immunogenic, especially those generated from cell lines grown in vitro for EV production [169].

## 12. Engineering EVs for Precision Delivery

Accumulating studies demonstrate that EVs can be functionally engineered to improve their disease-specific targeting and therapeutic efficacy. A variety of approaches, including enzymatic remodeling, liposome fusion, genetic modification, chemical functionalization, hydrophobic insertion, and metabolic engineering, have been developed to improve EV performance. To improve EVs’ in vivo effectiveness and lessen negative effects, surface modification can further increase their targeting capability. A wide range of strategies has been developed to engineer the surface of EVs, including genetic modification, click chemistry, metabolic labeling, affinity-based approaches, enzymatic ligation, lipid insertion, hydrophobic insertion, receptor–ligand interactions, and multivalent electrostatic binding. These approaches are commonly employed to enhance EV targeting efficiency [170,171].

More work needs to be carried out to increase the designed EVs’ freight loading efficiency. A key component of this novel cell-free therapeutic strategy is the development of improved methods to maximize cargo packaging efficiency, thereby reducing the required dose and frequency of administration of engineered EVs [172]. Thirdly, the usage of modified EVs may result in safety concerns because the contents and surface compositions of EVs may alter dramatically during modification operations [173]. Therefore, in order to prevent any negative effects, the use of modified EVs requires thorough preclinical testing. Overall, the development of engineered EVs offers fresh perspectives on the effective and focused administration of medicinal substances for refractory diseases [171].

## 13. Challenges for Implying EVs in Clinical Investigations

Clinical research on EVs, particularly exosomes, has garnered substantial attention due to their strong potential in early tumor detection and accurate cancer diagnosis. Current research emphasizes the identification of cancer-specific biomarkers within exosomes, offering valuable insights for precise disease classification. The isolation and molecular profiling of exosomes from cancer patients have revealed distinct cancer-associated signatures. These findings highlight the ability of exosomes for early diagnosis, enable timely therapeutic intervention, and improve clinical outcomes and prognosis. An important focus of ongoing research involves utilizing exosomal biomarkers to differentiate between various cancer types by creating multi-marker panels that improve diagnostic precision. Simultaneously, research assessing the predictive importance of exosomal profiles has connected particular vesicle signatures to clinical outcomes and disease development, supporting therapeutic choices. The clinical utility of exosome-based diagnostic techniques is being expanded by ongoing research into a variety of cancers [174].

**Figure 7:**
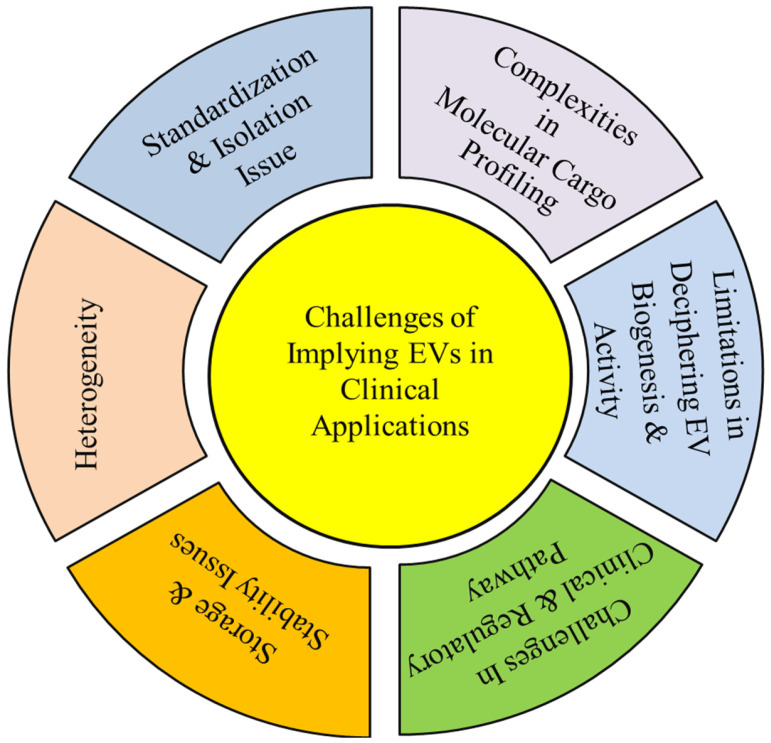
Challenges for utilizing EVs in clinical settings. This figure provides an overview of the principal biological, technical, and regulatory challenges that hinder the implementation of extracellular vesicle (EV)-based strategies in clinical applications.

Despite their considerable promise as biomarkers and therapeutic tools, the clinical translation of EVs remains at an early stage and is constrained by technical, methodological, and regulatory challenges. Although their involvement in diverse physiological and pathological processes supports their potential utility in disease diagnosis, prognosis, and therapy, issues related to isolation, characterization, standardization, and scalability continue to limit widespread clinical implementation [175] (Figure 7).

### 13.1. Standardization of Characterization and Isolation Procedures

Modern EV isolation approaches frequently co-isolate extracellular components, lipoproteins, protein aggregates, and other non-EV particles. The precision and specificity of downstream analyses are jeopardized by such impurities. Furthermore, variability in these isolation protocols contributes to substantial variations in EV yield, purity, and composition. This limits the reliability and reproducibility of EV-based biomarkers [175].

Hence, there is a growing need for standardized pre-analytical and analytical workflow to address these issues. Ideally, EV analysis should minimize extensive isolation steps and prioritize simpler, faster, and more integrated workflows. Liquid biopsy has emerged as a novel approach for EV detection from clinical samples, supported by electrochemical biosensor platforms like iMEX [32]. Notably, EV-associated DNA analysis has demonstrated improved sensitivity for detecting oncogenic mutations, including BRAFV600E, compared with cfDNA-based methods [176].

Given the technical challenges associated with EV size, heterogeneity, and low refractive index, consensus guidelines for EV isolation, measurement, and reporting are essential. For ensuring analytical consistency and cross study comparability, a combination of characterization techniques is required [177].

### 13.2. Heterogeneity

EVs are highly heterogenous, varying in size, cellular origin, and molecular cargo. Their composition can change in response to stress, pathological conditions, and treatment interventions. This heterogeneity complicates the identification of disease-specific biomarkers, and necessities the precise isolation of relevant EV subpopulations. Additionally, both biological variability (e.g., patient sex, age, disease state) and technical variability (e.g., sample handling and processing) further compromise the reproducibility and reliability of EV-based investigations, underscoring the importance of rigorous control across pre-analytical and analytical workflows [175].

In particular, distinguishing T-EVs from the background host of isolated EVs remains difficult, which makes accurate biomarker measurement challenging and slows their translation to clinical practice [178]. Identifying a suitable and validated housekeeping gene for EV normalization due to the pronounced EV heterogeneity is still a major difficulty. As a result, the clinical validity of T-EV-based biomarker studies depends on the careful selection and validation of reference genes that are appropriate for specific EV populations and biological sources [179,180].

### 13.3. Current Limitations in Deciphering EV Biogenesis and Activity

Even though research on EVs has advanced significantly, little is known about the mechanisms driving EV biogenesis, release, and uptake. Effective EV modification for the identification of biomarkers and therapeutic applications is hampered by our incomplete understanding of these mechanisms. The formation of EV-based biomarkers cannot proceed without a better understanding of EV biology [175]. Preclinical models have been used to evaluate a number of strategies. While GW4869, a pharmacological inhibitor of neutral sphingomyelinase 2 (nSMase2) involved in ceramide biosynthesis, reduces exosome secretion in oligodendroglial cells, and sphingolipid ceramide promotes exosome biogenesis by driving intraluminal vesicle formation and exosome release within MVBs [181].

Utilizing HEK293 and COS-7 non-cancer cells, Kosaka et al. reported utilizing GW4869 to limit the production of exosomal cargo, such as proteins and miRNAs, without substantially changing the exosome composition [182]. In vivo, GW4869 is utilized to inhibit EV secretion, particularly from tumor cells. While the intra-tumoral injection of GW4869 reduces tumor development, cancer-cell-derived EVs have been demonstrated to increase tumor growth in a colorectal cancer model by stimulating cancer cell proliferation and suppressing apoptosis [183].

### 13.4. Complexities in Molecular Cargo Profiling of EVs

Since EVs are complexes of multiple molecules, diagnostics can target any one of them. As more and more targetable molecules in EVs, like proteins or miRNAs, are discovered, the body of data will probably lead to the discovery of consensus EV markers [184].

Despite growing recognition of the diverse biological roles of exosomes, the molecular mechanisms governing the selective sorting and packaging of cargo into these vesicles remain poorly understood. It is still difficult to differentiate microvesicles from exosomes because there are no particular markers available [185]. Advanced and frequently sophisticated methods like next-generation sequencing and mass spectrometry are necessary for the analysis of EV cargos. Although they are constrained by existing technical capabilities, the sensitivity and specificity of analyses are essential for locating putative biomarkers. Better techniques are therefore required for high-throughput and high-sensitivity analyses [175].

The diversity of proteins regulating exosome formation and cargo selection complicates the development of flexible methods for loading biotherapeutic cargo into exosomes, even after much research into endogenous scaffolds for exosomal cargo engineering. Crucially, loading efficiency is significantly impacted by the cargo fusion partner selection. It is important to keep in mind that combining a particular cargo with one exosome biogenesis regulator could lead to less than ideal loading into exosomes, whereas combining it with another could produce better engineering performance [185].

### 13.5. Challenges in the Clinical and Regulatory Pathway of EV-Based Application

Numerous studies have identified new EV-based biomarkers; however, both general issues that are typical of the biomedical research area and particular issues that are unique to the nanoparticle field have hindered the technical and clinical validation phase. As a result, the biomarker discovery pipeline has seen more failures than successes. Therefore, in order to translate and apply new EV-based research to patient treatment, greater focus needs to be placed on the biomarker development, validation, and verification process [186]. Setting up platforms for the clinical grade production of EVs that meet all requirements for the successful approval of subsequent EV-based clinical trials is still difficult, especially when combined with the need to address a number of manufacturing and safety concerns as well as the implementation and validation of suitable quality controls. However, as a result of the significant advancements in EV research, better and standardized procedures for EV isolation and storage, along with enhanced approaches, strategies, and standards for evaluating the grade of EV-based treatments, need to be accessible shortly [187].

Navigating intricate regulatory networks is necessary to translate EV-based biomarkers into clinical practice. It is difficult and necessitates substantial clinical validation to guarantee the clinical efficacy, safety, and reproducibility of EV-based diagnostics and treatments [188,189]. The absence of regulatory criteria tailored to EV-based goods may cause a delay in their clinical implementation [190]. In addition, successful clinical translation of EV-based biomarkers will require scalable isolation and analytical workflows capable of handling large sample volumes efficiently. The creation of automated, high-throughput technology capable of processing clinical samples effectively and consistently is necessary for scalability [175].

### 13.6. Storage and Stability Issues

Even though EV vaccines are relatively easier to store and transport than cell-based therapies, this advantage is relative, as standardized long-term preservation strategies to maintain the stability and bioactivity of EVs remain a significant challenge. This is a crucial subject for additional research because it is yet unknown how stable EVs are during storage and how storage circumstances affect EV integrity and bioactivity. When EV-based biomarkers are transported or stored for long periods of time, this uncertainty may compromise their dependability. To preserve the integrity of EVs, consistent storage techniques must be developed [175]. According to recent research, the yield, integrity, amount, contents, and functionality of EVs are significantly impacted by storage conditions [191]. Repeated freeze–thaw cycles can further jeopardize EV stability, and storage effects vary based on sample origin and handling conditions [191,192]. Although −80 °C is now thought to be the most practicable choice for long-term storage, there are still issues with its cost and logistical constraints [191,192]. Storage at 4 °C is suitable for short-term handling, but more research is required to develop standardized long-term storage and cryopreservation techniques to guarantee EV integrity, functionality, and reproducibility [191,192]. As a long-term EV storage solution, liquid nitrogen has been investigated. Even in the absence of cryoprotectants, rapid freezing techniques like snap freezing or vitrification better retain EV particle counts than slow freezing. However, membrane damage can be caused by both liquid nitrogen and −80 °C storage, with some data indicating that liquid nitrogen conditions cause more disruption. RNA integrity is partially preserved by controlled cooling rates (~−1 °C/min), underscoring the necessity of optimizing freezing techniques and using suitable cryoprotectants to retain EV stability and functionality [193].

### 13.7. Ethical and Regulatory Challenges Associated with EV-Based Therapeutics

Significant ethical and regulatory obstacles prevent EV-based cancer treatments from being widely used in clinical settings. The absence of scalable, high-yield purifying techniques, difficult biogenesis, and EV heterogeneity impede large-scale production with consistent quality and potency. Batch-to-batch consistency cannot be guaranteed by current quality control and standardization procedures that were created for research purposes. EV therapies lack established frameworks, making regulatory compliance difficult because organizations like the FDA want thorough documentation of manufacture, safety, and efficacy. Donor cell source, the possible transfer of immunomodulatory or carcinogenic cargo, and the safe use of modified EVs are all ethical issues. To enable the safe and efficient clinical implementation of EV-based medicines, these difficulties underscore the critical requirement for consistent production, strong quality control, and unambiguous regulatory guidance [194].

## 14. Role of EVs Beyond Metastasis

While the role of EVs in metastatic-dissemination has been extensively investigated, accumulating evidence indicates that EVs also play critical roles in tumor initiation, progression, and the development of therapy resistance, thereby influencing multiple stages of cancer progression. T-EVs may carry oncogenic proteins, RNA, and DNA with the potential to reprogram recipient cells. The EV-mediated transfer of oncogenic cargo may induce phenotypic changes associated with malignant transformation and stromal reprogramming. These observations have led to the concept of horizontal transformation or genometastasis, whereby EVs contribute to tumor initiation and progression [195].

Beyond DNA and protein cargo, EV-mediated communication supports tumor growth and metastasis through the transfer of RNA cargoes including mRNA and non-coding RNA (ncRNAs) that regulate crosstalk within TME and premetastatic niches. These EV-associated RNAs modulate key cancer hallmarks and drive tumor progression [196]. Emerging evidence also highlights the role of exosomal nc-RNAs in microenvironment remodeling across diverse cancers, including sarcomas, where they influence proliferation, invasion, angiogenesis, and immune interactions. Additionally, they hold promise as translational biomarkers and therapeutic targets [197]. EVs also contribute to horizontal transfer of drug resistance by delivering bioactive cargo that reprograms gene expression and signaling pathways in recipient cells. EVs derived from drug-resistant cancer cells can induce resistance in sensitive cells by transferring resistance-associated proteins and regulatory molecules, including Nrf2, P-glycoprotein, GSTP1, and activated STAT3. Such EV-mediated cargo transfer enhances cancer cell survival under chemotherapeutic treatment, highlighting EVs as important mediators of acquired therapy resistance [198].

## 15. Tumor Type Specific Roles of EVs Across Various Cancers

Accumulating evidence indicates that EVs exert tumor-type-specific functions in cancer progression, reflecting differences in cellular origin, microenvironmental context, and molecular cargo. Across diverse malignancies, EVs mediate distinct mechanisms that influence tumor growth, metastasis, immune modulation, and therapy response, underscoring their context-dependent roles in cancer biology.

### 15.1. Breast Cancer

By transferring oncogenic payloads that remodel recipient cells in both local and distant microenvironments, EVs produced from breast cancer aid in the progression of metastases. In order to support tumor cell survival and dissemination, these EVs stimulate EMT, improve angiogenesis and vascular permeability, inhibit antitumor responses, and alter stromal habitats. Organ-specific tropism and treatment resistance are supported by genetic cargo in EVs, such as mutant transcripts and miRNAs linked to metastasis. When taken as a whole, breast cancer EVs function as dynamic intercellular communication mediators that promote metastatic colonization and serve as therapeutic targets and biomarkers [199,200,201].

### 15.2. Gastric Cancer

EVs frequently interact with GC metastasis through the many pathways. First, by releasing bioactive chemicals, EVs can influence cell contacts and signal transduction in TME, hence controlling the GC metastatic process. Second, the molecules released by EVs, namely non-coding RNAs and proteins linked to metastasis, can control GC cells’ EMT and MMT and increase their capacity to spread. Additionally, immunoregulatory chemicals found in EVs can control immune suppression and tumor immune escape, which can impact GC cell metastasis as well as immune identification and clearance. Finally, the bioactive compounds in EVs have the ability to control vascular growth factor expression, which influences angiogenesis. The newly created blood arteries improve GC cells’ capacity to spread as well as their blood supply and nutrition [202].

### 15.3. Pancreatic Cancer

EVs are essential in pancreatic cancer because they support angiogenesis and chemoresistance, promote cancer cell proliferation and survival, impede immune response, and facilitate cell–cell communication. The unique oncogenic makeup of cancer-derived EVs promotes the growth and spread of tumors. They are therefore important potential biomarkers for a number of malignancies, including pancreatic cancer. Notably, EVs are a perfect minimally invasive diagnostic and monitoring tool for pancreatic cancer patients since they can be separated from a variety of biological fluids, including blood, urine, and saliva [203].

### 15.4. Liver Cancer

T-EVs act as key mediators of systemic hepatic reprogramming in cancer. They induce inflammation, fatty liver formation, and metabolic dysregulation even in the absence of liver metastasis. EV cargo activates Kupffer cells to secrete TNF, creating a pro-inflammatory hepatic microenvironment that suppresses fatty acid metabolism, oxidative phosphorylation, and cytochrome P450 expression. This EV-driven liver dysfunction impairs drug metabolism and increases chemotherapy toxicity, highlighting tumor EVs as critical regulators of liver physiology and potential therapeutic targets to improve treatment tolerance and efficacy [204]. EVs play an important role in regulating HCC metastasis, immune evasion, and proliferation. Through the transfer of oncogenic miRNAs, proteins, and non-coding RNAs that alter both tumor and stromal cells, HCC-derived EVs function as important mediators of tumor progression by encouraging EMT, metastasis, immune evasion, and post-treatment recurrence. Additionally, EVs are gaining interest as a desirable biomarker for HCC identification because they are seen in the bloodstream at comparatively early stages of the disease. EVs are therefore thought of as possible therapeutic agents or vehicles for the treatment of HCC. Functional proteins, nc-RNAs, and mRNAs carried by EVs are promising biomarkers for the identification of early-stage HCC, according to new research in the field [205].

### 15.5. Melanoma

Melanoma cells release EVs with a diverse cargo that includes proteins, mRNA, miRNA, non-coding RNAs, DNA, lipids, and metabolites. Melanosomes, exosomes (Exos), microvesicles (MVs), and extracellular particles (EPs) are among the EVs secreted by melanoma cells. Secreted vesicles affect the phenotypic behavior of many immunological and stromal populations inside the tumor microenvironment. Melanoma EVs primarily cause immunological tolerance, angiogenesis, and CAF development in the tumor microenvironment. Additionally, EVs encourage phenotypic alterations in the lymph nodes that drain tumors, the bone marrow, and distant organs like the lung, helping to create an effective pre-metastatic niche that enables melanoma cells to colonize distant organs [206].

### 15.6. Lung Cancer

Every stage of lung cancer metastasis involves EVs produced by tumor cells and other TME cells. EVs contain proteins and RNAs that can cause angiogenesis and EMT. Furthermore, by activating Treg, changing the characteristics of DC, suppressing the activity of T cells and natural killer (NK) cells, and causing the polarization of macrophages (MCs) from type 1 to type 2, EVs are linked to controlling inflammatory responses in the TME, in circulation, and at distant metastatic sites. By altering the pre-metastatic niche environment, EVs contribute to vascular permeability and organotropism at remote locations. For each step, significant EV content that has been linked to lung cancer metastasis is indicated [207].

### 15.7. Prostate Cancer

By encouraging tumor growth, invasion, bone metastasis, and medication resistance, EVs play a crucial role in the formation of the prostate tumor microenvironment. By altering the stroma, prostate cancer EVs facilitate the development of a tumor-supportive environment. Furthermore, they trigger fibroblast activation, which raises EV shedding and triggers prostate cancer cell migration and invasion through the CX3CL1-CX3CR1 pathway. The elevated levels of ITGA3 and ITGB1 in urine EVs from patients with metastatic prostate cancer can be explained by the fact that prostate cancer-derived EVs also cause the migration and invasion of prostate epithelial cells via integrin α3 (ITGA3) and integrin β1 (ITGB1). Through the transforming growth factor β (TGFβ), prostate cancer EVs stimulate fibroblast differentiation to a pro-tumorigenic phenotype, resulting in angiogenesis and faster tumor growth [208].

### 15.8. CRCs

By controlling the tumor cells’ microenvironment, EVs can control the growth, metastasis, and infiltration of CRC cells as well as intercellular communication between CRC cells and target cells. EVs are anticipated to function as novel molecular markers for cancer diagnosis since they carry particular molecular components in source CRC cells. EVs display a pro-angiogenesis role in CRC via the miR-221-3p pathway [209].

### 15.9. OSCCs

Tumor-derived exosomes (TDEs) have the ability to enhance invasion and migratory capabilities by promoting EMT. Numerous studies have demonstrated that exosomal miRNAs can contribute to oncogenesis and that CAFs can interact with tumor cells through exosomes. The interaction between EVs and tumor cells is a bidirectional regulation process that varies significantly depending on the components of EVs. For example, fibroblasts may contribute to OSCC progression through the AKT/GSK-3β/β-catenin/Snail signaling pathway and transfer exosomal miRNA-34a-5p to OSCC cells. Such results are influenced by the components of exosomes, drug efflux by EVs, vesicular pH changes, the anti-apoptotic signal transmitted by EVs, immunological response, DNA repair mechanism modulation, and EV-induced cancer stemness and EMT [210].

## 16. Functional Roles of Extracellular Vesicle Subtypes in Tumor Progression and Metastasis

Different EV subtypes are increasingly recognized as having distinct characteristics and functional roles in cancer due to differences in their biogenesis, size, and molecular cargo composition. Exosomes, which originate from the intraluminal vesicles of MVBs, carry a broad spectrum of proteins, lipids, and nucleic acids that can modulate gene expression, immune responses expressions, angiogenesis, invasion, and metastatic progression in recipient cells, making them key mediators of intercellular communication in tumors [98].

Because exosomes facilitate intercellular communication both inside the TME and at distant organ locations, exosomes are essential for cancer metastasis. Tumor-derived exosomes facilitate important stages of the metastatic cascade by transferring bioactive cargo, such as proteins, lipids, and nucleic acids, between cancer cells, stromal cells, endothelial cells, and immune cells. These vesicles promote angiogenesis and vascular permeability, aid in EMT and extracellular matrix remodeling, and inhibit antitumor immunological responses, allowing tumor cells to elude immune monitoring. Furthermore, by reprogramming distant tissues to facilitate tumor cell colonization and survival, exosomes promote the creation of pre-metastatic habitats. Exosomes have an active and complex role in promoting tumor spread and metastatic progression, as evidenced by their ability to identify organ-specific metastatic tropism through distinct molecular fingerprints on their surface [211].

Tumor-derived MBs contain oncogenic proteins, cytoskeletal regulators, proteases, and signaling molecules that promote the invasion and migration of tumor cells. MBs promote extracellular matrix remodeling and local tumor invasion by transporting matrix-degrading enzymes such metalloproteinases and invasion-associated signaling complexes. Additionally, by delivering bioactive cargo that encourages angiogenesis, vascular permeability, and immunological evasion, MBs alter the behavior of stromal, endothelial, and immune cells. These vesicles promote tumor cell intravasation and dissemination and help create a supportive tumor microenvironment [212,213].

By delivering useful biomolecules to surviving tumor cells, apoptotic bodies generated by dying tumor cells can actively increase cancer malignancy and metastatic behavior. EVs formed from apoptotic cells transfer splicing factors to receiving cancer cells, as seen in glioblastoma. This results in extensive changes in RNA splicing that increase tumor aggressiveness, invasion, and adaptability. Instead of serving as inert byproducts of cell death, apoptotic bodies contribute to tumor heterogeneity and allow surviving cancer cells to develop more malignant phenotypes through this intercellular transfer of regulatory proteins and nucleic acids. This facilitates tumor progression and metastatic potential [214].

## 17. Conclusions

EVs have been recognized as key modulators of cancer progression due to their multifaceted roles in intercellular communication, TME modulation, immune regulation, and metastatic niche formation. Advances in EV biology have highlighted their significant potential as minimally invasive biomarkers for early cancer detection, prognosis, and therapeutic response monitoring, as well as promising platforms for targeted drug delivery. Despite these encouraging attributes, challenges related to standardized isolation methods, cargo heterogeneity, scalability, and clinical validation continue to limit their broad clinical translation. Addressing these limitations through technological innovation and large-scale, well-designed clinical studies will be critical for integrating EV-based strategies into routine oncology practice. Continued interdisciplinary research is expected to accelerate the incorporation of extracellular vesicles into precision cancer medicine.

## Figures and Tables

**Table 1:** Mechanistic role of extracellular vesicles in TME.

Mechanisms	Extracellular Vesicles Source and Cargo (miRNA/Protein)	Target/Effect	Implications	References
Angiogenesis	Tumor derived exosomal matrix metalloproteinases (MMP-2, MMP-9, MT1-MMP)	Local extracellular matrix (ECM) degradation, supports angiogenesis and tissue remodeling	Role in angiogenesis, wound repair, tumor invasion, potential therapeutic targeting	[61]
Immunomodulation	Plasma derived carrying ARG1/CD3/PD-L1/PD-L2	Stronger antitumor immunity microenvironmental characteristics, such as more activated CD8+ T/NK cells, a greater TH1/TH2 ratio, and higher expressions of IFN-γ/perforin/granzymes, were represented in a high EV-score	While EV-score < 1 GC might have benefited more from ICIs combining HER2-targeted therapy, EV-score ≥ 1 GC obtained more therapeutic benefits from ICIs	[62]
Fibroblast modulation/ECM remodeling	Exosomes derived from fibroblasts carrying tumor-targeting proteins, microRNAs (miRs)	Highly efficient for pancreatic cancer	Improving personalized therapeutics and tumor targeting drug delivery vehicles	[63]
Fibroblasts modulation and immunomodulation	GLUT1 inhibitor	Tumor glycolysis, fibrotic ECM stiffness, Programmed death-ligand 1/Programmed death-1 Receptor (PD-L1/PD-1) immune check point, enhanced tumor localization and matrix penetration	Suppress tumor glucose metabolism, remodel fibrotic ECM, enhance response to PD-L1 immunotherapy,	[64]
Immunomodulation	Endothelial cells derived from endothelial cells carrying pro-inflammatory molecules	EVs increased monocyte penetration of the endothelium and encouraged monocyte accumulation. The inflammatory polarization of macrophages from the M2 to the M1 phenotype was triggered by these EVs	EVs contribute to early-stage atherosclerosis, MAPK inhibition may offer targeted therapeutic intervention	[65]
Immunomodulation	Vascular endothelial cells derived and carrying pro-inflammatory molecules, adhesion molecules, and signaling mediators	Induce proinflammatory activation in HUVECs and mixed response in monocytes, promote adhesion and migration	Enhancement of vascular inflammation, contributing to vascular diseases and immune driven pathology	[66]
Diagnostic biomarker	Endothelial cells derived and having EV surface protein, endothelial dysfunction-related biomarkers	Reflects endothelial cell health, used as a circulating foot print for INOCA	Potential non-invasive marker for diagnosing INOCA and distinguishing its endotypes, may streamlines diagnostic algorithms	[67]
Clinical translational aspect/Therapeutic application	Allogenic adipose mesenchymal stromal cells derived and carrying anti-inflammatory and regulatory molecules including protein, RNAs, lipids	By reducing lung inflammation and histopathological severity, nebulizing haMSC-EVs increased the survival rate	Preclinical efficacy in models of lung injury/ARDS, clinical safety shown in early trials with nebulized EVs, suggests strong potentials for cell-free therapy in respiratory diseases including ARDS and possibly COVID-19	[68]
Pre-metastatic niche	Melanoma-derived exosomes enriched for receptor tyrosine-kinase	Reprogram bone marrow progenitors to a pro-vasculogenic, pro-metastatic phenotype	Systemic niche priming that accelerates metastasis	[69]
Organotropic metastasis	Exosomal integrins α6β4 and α6β1 linked to lung metastasis, exosomal integrin αvβ5 linked to liver metastasis	Engage organ-specific resident cells (e.g., fibroblasts/epithelial cells; liver Kupffer cells), activate Src/S100 pathways	Organ-specific metastases could be predicted using exosomal integrins	[70]
Pre-mechanistic niche	Melanoma-derived exosomes enriched in CD3-associated proteins and metabolic regulators	Reprograming distant stromal and bone marrow-derived cells to support metastatic dissemination	Tumor-permissive pre-metastatic niche via CD36.	[71]
Metabolic reprograming	Breast cancer exosomal miR-122	Suppresses glucose uptake in niche cells	Enhance metastatic colonization	[72]
Vascular barrier disruption	Breast cancer exosomal miR-105	Targets ZO-1 in endothelial cells, breaks tight junction, increases permeability	Facilitates intravasion/Extravsion and metastasis	[73]
Immunosuppression	Tumor-derived PD-L1	Triggers the immunological checkpoint response by interacting with T cells’ programmed death-1 (PD-1) receptor	The amount of circulating exosomal PD-L1 in patients with metastatic melanoma varies throughout anti-PD-1 therapy and has a favorable correlation with that of IFN-γ	[74]
ECM remodeling	EVs derived from bone marrow mesenchymal stem cells (BM-MSCs) carrying Pro-regenerative and proteolytic signals, procrine modulators like proteins, lipids, RNAs	Reducing the proteolytic activity and providing benefits for the regeneration of elastic matrix in an aneurysmal setting	An explanation of the significance localized, rupture-prone aortic aneurysms (AAAs) are caused by the enzymatic breakdown of elastic fibers, which reduces the aorta’s wall flexibility	[75]
Metastasis (Epithelial–mesenchymal transition or EMT induction)	Melanoma cell–derived exosomes carrying miR-191 and let-7a	Through paracrine/autocrine signaling, exosomes produced from melanoma cells encourage phenotypic flipping in primary melanocytes	Exosomes encourage the tumor microenvironment’s EMT-like process	[76]

**Table 2:** Clinical Relevance of EVs in Cancer Diagnosis and Prognosis.

Cancer Type	EVs Source	Key Cargo	Clinical Utility	Reference
NSCLC	Plasma	Immunosupressive molecules e.g., CD39, CD73, PD-1, PD-L1, CTLA-4, TGFβ, Fas, FasL, and COX-2; Immunostimulatory protein “OX40L’	Pre-therapy plasma-derived small EVs (sEV) may be helpful as non-invasive biomarkers of clinical outcome and therapy response in NSCLC.	[119]
Early lung cancer	Plasma	CD81, PD-L1, GLIPR1, LBR, SFTPA1, EVELC-M5	According to this study, EVELC-M5 has a lot of potential for clinical application and is a useful diagnostic tool for identifying early lung cancer.	[120]
NSCLC	Plasma	CD9, CD63, CD81, Surface proteins	With just 10 µL of unpurified plasma, the EV array analysis was able to identify and characterize exosomes in every sample.	[121]
Liver fibrosis	Serum	miR-34c, -151-3p, -483-5p or -532-5p	Serum EVs from healthy, normal people are naturally anti-fibrogenic and anti-fibrotic. They also contain microRNAs that can help recover injured hepatocytes or activated HSC.	[122]
Hepatocellular carcinoma	Plasma	mRNAs, circRNAs, lncRNAs	This study found that human plasma had a large amount of extracellular vesicle long RNA (exLR) and discovered a variety of distinct indicators that may be helpful in the detection of cancer.	[123]
Oral squamous cell carcinoma (OSCC)	Plasma	CD63, CAV-1	A longer life expectancy for OSCC patients was associated with decreased levels of plasmatic exosomes both prior to and following surgery.	[124]
OSCC	Saliva	PPlA, MMP-9, S1A009, Myosin, Rab GD1	The study shows that saliva-derived EVs carry proteins that differ significantly between OSCC patients and healthy controls.	[125]
Prostate cancer	Plasma	CD63, Prostrate-specific membrane antigen (PSMA), caveolin-1	Plasma exosomal PSMA and caveo-lin-1 serve as liquid biopsy biomarkers for diagnosis and prognosis of aggressive prostate cancer	[126]
Prostate cancer	Urine	MiR-19b, miR-25, miR-125b, and miR-205	The 100%/93% and 95%/79% specificity/sensitivity of miR-19b against miR-16 detection in total vesicles and exosome-enriched fractions, respectively, demonstrates the difficulty in differentiating cancer patients from healthy persons.	[127]
Brain metastasis	Plasma	Pd-L1, pSTAT3α	Patients with brain metastases from melanoma are identified by plasma circulating sEVs showing elevated PD-L1 and decreased STAT3 activity.	[128]
Clear cell renal cell carcinoma (ccRCC)	Plasma	TIMP-1 mRNA, TIMP-2 mRNA, MMP-1 mRNA	For ccRCC, EV-derived TIMP-1 mRNA might be a promising predictive biomarker candidate.	[129]
ccRCCs	Urine	CD63, CD9, and CD81, CA9	Because exosomes carry lipids, RNA, and tumor proteins, they have emerged as a significant source for liquid biopsies. The most practical biological liquid for exosome sampling is urine.	[130]
Melanoma	Plasma	BRAFV600E	Mutant DNA interacts either directly with the peptide or with the outside side of the EV membrane, leaving it mostly vulnerable to nuclease digestion.	[131]
Melanoma	Plasma	Proteins (APOC4, PRG4, PLG, TNC, VWF and SERPIND1) and metabolites (lyso PC a C18:2, PC ae C44:3)	The ability of coupled proteo-metabolomic signatures to distinguish across disease phases may offer important information about prognosis, early detection, and individualized therapy plans.	[132]
Gastric cancer	Gastric juice	CagA, VacA proteins	They can cause macrophages to produce interleukin (IL)-6 and IL-1β, gastric epithelial cells to produce IL-8, and tumor necrosis factor-α to be produced.	[133]
Gastric cancer	Plasma	ARG1, CD3, PD-L1, PD-L2	High EV-score links to stronger antitumor immunity and better clinical benefit.	[134]
CRC	Plasma	CD133+, EPCAM+	Compared to healthy controls, patients with advanced colorectal cancer have greater blood levels of total, CD133+, and EPCAM+ EVs, suggesting that the tumor-induced phenotypic alterations are responsible for this rise.	[135]
CRC	Stool	Bacterial taxa profile	Microbe-derived EV profiling might provide a new biomarker for identifying and forecasting the prognosis of colorectal cancer.	[136]

## Data Availability

No new data were created or analyzed in this study.

## Abbreviations

The following abbreviations are used in this manuscript:

A2780 cells	Human ovarian cancer cell line
A2780/DDP	Cisplatin-resistant derivative of human ovarian cancer cell line
ARF6	ADP-ribosylation factor 6
ARG1	Arginase-1
BBB	Blood–brain barrier
BM-MSCs	Bone marrow mesenchymal stem cells
CAFs	Cancer-associated fibroblasts
CagA	Cytotoxin-associated gene A
Cav-1	Caveolin-1
CD3	Cluster of differentiation 3
CD39	Cluster of Differentiation 39
CD73	Cluster of Differentiation 73 (also known as Ecto-5′-nucleotidase)
CD133+	Cluster of Differentiation 133 (positive expression)
CNS	Central nervous system
COX-2	Cyclooxygenase-2
CRC	Colorectal cancer
CTL	Cytotoxic T lymphocytes
CTLA-4	Cytotoxic T-Lymphocyte Associated Protein 4
DCs	Dendritic cells
DEX	Dendritic cell-derived exosomes
DLS	Dynamic light scattering
ECM	Extracellular matrix component
EMT	Epithelial–mesenchymal transition
EPCAM+	Epithelial Cell Adhesion Molecule (positive expression)
EVELC-M5	Extracellular Vesicle Early Lung Cancer membrane protein 5
EVs	Extracellular vesicles
EVX-M+P	Extracellular vesicles-based combined mRNA and protein vaccine platform
Fas	Fas receptor (also known as CD95)
FasL	Fas ligand (also known as CD95L)
GBM	Glioblastoma
GAG	Group-specific antigen
GC	Gastric cancer
gDNA	Genomic DNA
GET	Gene editing tools
GLIPR1	Glioma Pathogenesis-Related Protein 1
ILVs	Intraluminal vesicles
LBR	Lamin B Receptor
LC	Lung cancer
LCMV	Lymphocytic choriomeningitis virus
Lung-Exos	Lung-derived exosomes
lncRNAs	Long non-coding RNAs
MDSCs	Myeloid-derived suppressor cells
MDV	Marek’s disease virus
MIF	Migration inhibitory factor
miR-25	MicroRNA-25
miR-125b	MicroRNA-125b
miR-205	MicroRNA-205
MMP	Matrix metalloproteinases
mRNA	Messenger RNA
miRNA	Micro RNA
MSCs	Mesenchymal stem cells
MVs	Microvesicles
MSC-EVs	Mesenchymal stem cell-derived stem cells
MVBs	Multivesicular bodies
nSMase2	Neutral sphingomyelinase 2
OSCC	Oral squamous cell carcinoma
OX40L	OX40 Ligand (also known as CD252)
OVA-Pulsed DEXs	Ovalbumin-pulsed dendritic cell-derived exosomes
PDACs	Pancreatic ductal adeno-carcinomas
PD-L1	Programmed death-ligand 1
PD-1	Programmed death-1 receptor
PEG	Polyethylene glycol
PMN	Pre-metastatic niche
PPIA	Peptidyl-prolyl cis-trans isomerase A (also known as Cyclophilin A)
PRRSV	Porcine reproductive and respiratory syndrome virus
Rab GD1	Rab GDP-dissociation inhibitor 1
RCC	Renal cell carcinoma
S100A9	S100 calcium-binding protein A9
SACC	Salivary Adenoid Cystic Carcinoma
SC	Skin cancer
SCLC	Small cell lung cancer
SFTPA1:	Surfactant Protein A1
S-Lipo	Spike mRNA-loaded liposomes
STAT3	Signal transducer and activator of transcription 3
T-EVs	Tumor-derived extracellular vesicles
TGFβ	Transforming Growth Factor-beta
TME	Tumor microenvironment
Tregs	Regulatory T cells
tRNAs	Transfer RNAs
VacA	Vacuolating cytotoxin A
